# Predicting Archaic Hominin Phenotypes from Genomic Data

**DOI:** 10.1146/annurev-genom-111521-121903

**Published:** 2022-04-19

**Authors:** Colin M. Brand, Laura L. Colbran, John A. Capra

**Affiliations:** 1 Department of Epidemiology and Biostatistics, University of California, San Francisco, California, USA; 2 Bakar Computational Health Sciences Institute, University of California, San Francisco, California, USA; 3 Department of Genetics, Perelman School of Medicine, University of Pennsylvania, Philadelphia, Pennsylvania, USA

**Keywords:** ancient DNA, archaic hominin, Denisovan, Neanderthal, phenotype prediction

## Abstract

Ancient DNA provides a powerful window into the biology of extant and extinct species, including humans’ closest relatives: Denisovans and Neanderthals. Here, we review what is known about archaic hominin phenotypes from genomic data and how those inferences have been made. We contend that understanding the influence of variants on lower-level molecular phenotypes—such as gene expression and protein function—is a promising approach to using ancient DNA to learn about archaic hominin traits. Molecular phenotypes have simpler genetic architectures than organism-level complex phenotypes, and this approach enables moving beyond association studies by proposing hypotheses about the effects of archaic variants that are testable in model systems. The major challenge to understanding archaic hominin phenotypes is broadening our ability to accurately map genotypes to phenotypes, but ongoing advances ensure that there will be much more to learn about archaic hominin phenotypes from their genomes.

## INTRODUCTION

Humans are the sole remnants of a hominin lineage that last shared a common ancestor with other extant apes 5–7 million years ago (92, 107, 133, 136). A rich paleontological record has revealed many species and populations belonging to this lineage, with some hominins co-occurring in time, and sometimes space, throughout the Plio-Pleistocene (66, 135). Both fossils and archaeological evidence have provided substantial insight into the phenotypes of some of these ancient individuals, particularly locomotor behavior (93) and diet (123). However, most traits cannot be inferred from available physical evidence, and this substantially limits progress in anthropology and evolutionary biology.

Over the past 15 years, the development of molecular and computational methods to sequence and analyze ancient DNA has presented a new opportunity to study ancient hominin populations (50, 113, 115). During the initial sequencing of ancient DNA from archaic humans and hominins, considerable effort has been devoted to identifying and tracing the movements of and interactions among ancestral groups (84, 90). Perhaps the most striking result from these studies is that interbreeding between humans and archaic hominins, like the Neanderthals, occurred and was likely common. We refer readers to excellent reviews on these topics (1, 2, 29, 31, 38, 96, 100, 129, 134). Approximately 40–50% of the Neanderthal genome can be reassembled from fragments found in modern human populations (105, 114), and at some loci Neanderthal ancestry was likely subject to purifying selection (48, 57, 87), while others may represent cases of adaptive introgression (43, 52, 102). This suggests that genomic differences underlie phenotypic differences between archaics and modern humans, even beyond those observable in skeletons. Connecting archaic genomes to phenotypes provides an opportunity to characterize similarities and differences between archaics and modern humans.

Here, we review how analyses of ancient DNA have begun to reveal glimpses of the phenotypes of archaic hominins. The genetic results extend, and in some cases complement, insights gained from fossils, but reconstructing the biology of ancient individuals from genetic and genomic data is still in its infancy and poses substantial challenges. We begin with a brief history of the evolution of these lineages and the available data from which we can infer phenotypes. Next, we evaluate our current understanding of archaic hominin phenotypes at levels from basic molecular biology to high-level and often complex phenotypes, such as behavior, health, and physical appearance. However, phenotypes at the highest level are incompletely understood in living humans and other model organisms, making their assessment in extinct groups even more difficult. In addition, because archaic hominins are extinct, directly evaluating the effects of variants by necessity must occur outside their true genomic context (i.e., not in a purely Neanderthal cell with a complete Neanderthal genome) and environment, which complicates interpretation of even experimental results.

**Figure 1:**
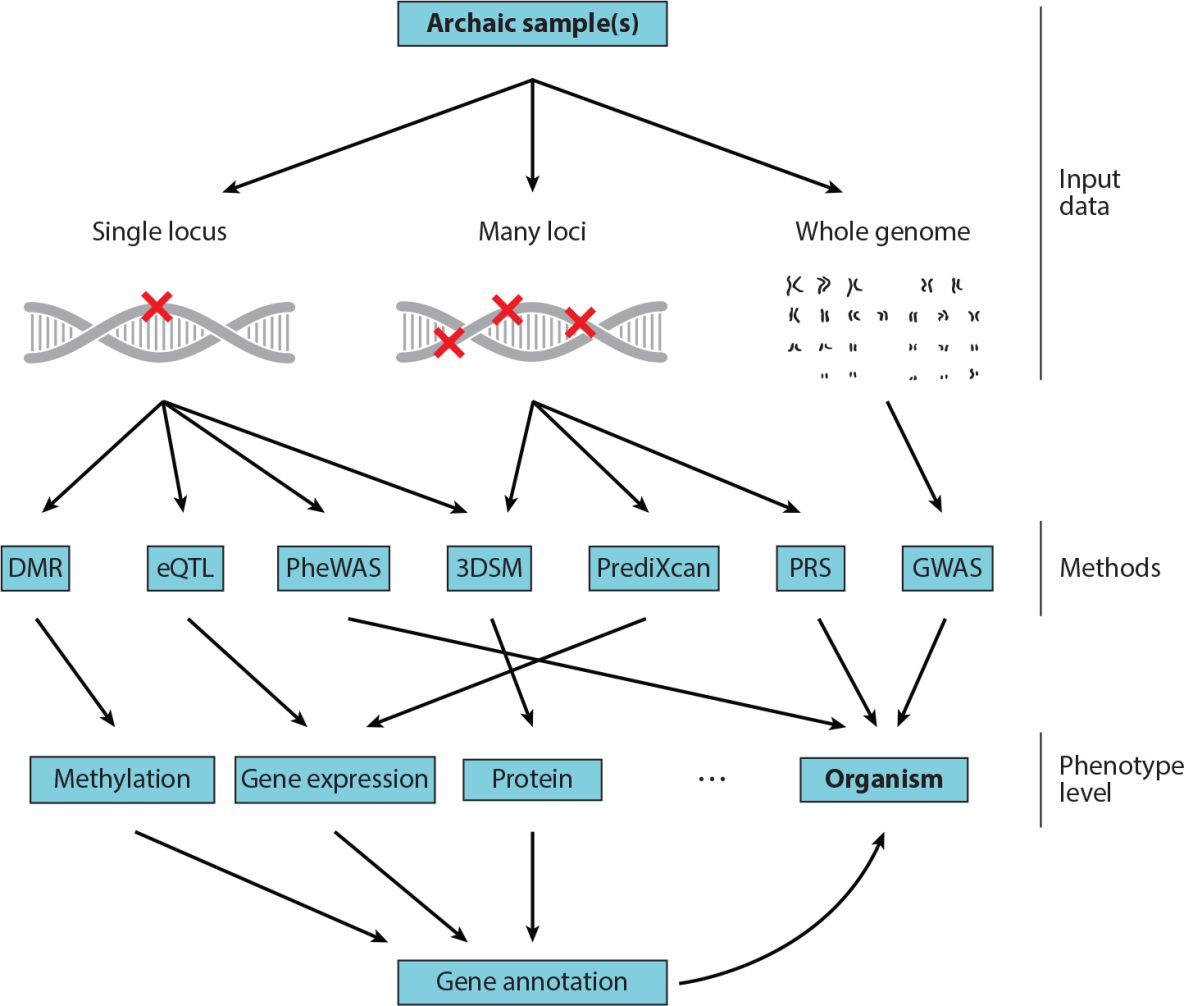
Techniques for understanding archaic phenotypes from ancient DNA. Various methods can be applied to different types of archaic input data—from individual variants to whole genomes—to infer multiple levels of phenotype. Several methods attempt to directly estimate organism-level phenotype from DNA sequence (GWAS, PheWAS, and PRS), whereas many others rely on predictions of molecular phenotypes with lower levels of biological complexity (methylation, gene expression, protein structure) paired with gene annotation to infer a variant’s impact on phenotype. Abbreviations: 3DSM, three-dimensional protein structural modeling; DMR, differentially methylated region; eQTL, expression quantitative trait locus; GWAS, genome-wide association study; PheWAS, phenome-wide association study; PRS, polygenic risk score.

We argue that progress in estimating phenotypes of ancient individuals will depend on increasing our understanding of the complex mapping between genotype and phenotype and how it is modulated by environmental context. Phenotypes occur at various levels of biological complexity, ranging from the molecular to the entire organism (Figure 1), and more complex phenotypes are more challenging to infer from genetic information than molecular phenotypes. Therefore, we propose that the most tractable approach to understanding the phenotypes of archaic hominins and other species is to focus on lower-level molecular phenotypes—such as gene expression levels, epigenetic states, or protein function—whose relationships to genotypes are better understood, less likely to vary across populations, and easier to evaluate in model systems. Lastly, we conclude with a discussion of outstanding questions and promising methods for future work.

## EVOLUTIONARY HISTORY OF THE DENISOVANS AND NEANDERTHALS

There is only limited fossil evidence for many early archaic hominins, and the availability of DNA or protein sequence data is even more limited. At present, the oldest sequences from human evolutionary history include dental proteomes from a *Homo erectus* specimen from Dmanisi, Georgia, dated to 1.77 Ma, and from a *Homo antecessor* specimen from Atapuerca, Spain, dated to 772–949 ka (132). While these sequences provide critical insight into the phylogenetic relationships of Pleistocene hominins, little can be inferred about the phenotypes of these taxa. Therefore, we focus our attention on the biology of two key Late Pleistocene hominins with genome-scale data: Denisovans and Neanderthals. There is a rich archaeological and fossil record for Neanderthals, while Denisovans are known almost entirely through their genome sequence.

The ancestors of Denisovans and Neanderthals likely emerged from Africa during the mid-Pleistocene, approximately 765–550 ka (95, 99). This population not only encountered novel environments but may also have encountered and interbred with at least one additional hominin species inhabiting Eurasia, the superarchaics (99). While the geographic range of the Denisovan–Neanderthal common ancestor is unknown, it is clear that they eventually experienced a bottle-neck and subsequently diverged into separate lineages. Estimates of when this divergence occurred range from 381 to 473 ka (94, 95), but it may be even older (>600 ka) (99).

The oldest known Neanderthals inhabited southern Siberia approximately 80–140 ka (32, 70, 94), although it is possible that this region was occupied as early as 193 ka (55). Neanderthals would later populate western Asia, the Middle East, and Europe until their extinction approximately 28 ka (56). However, at least one Neanderthal lineage remained in or returned to southern Siberia and was more closely related to non-Siberian than Siberian Neanderthals (70). Neanderthals likely interbred with newly arriving modern humans at least once, resulting in detectable Neanderthal ancestry in all populations descended from the out-of-Africa migration. Some Africans have lower levels of Neanderthal ancestry, likely due to migration back to Africa by non-Africans with Neanderthal ancestry and subsequent gene flow (18, 44, 103, 131). The slightly higher levels of Neanderthal ancestry in East Asians compared with Europeans was originally attributed to a lower effective population size in East Asia limiting purifying selection acting on deleterious introgressed variants (104). Alternatively, Neanderthal ancestry could be diluted in Europeans if they subsequently interbred with an unadmixed population (65, 80, 125). However, some have argued that these hypotheses are not well supported (57, 60) and that the differences in ancestry are better explained by multiple episodes of admixture (126, 128), but this remains an open question (11).

While Neanderthals are known from a rich fossil and archaeological record, physical remnants of Denisovans are scarce. Denisovans are known from three molars (97, 106, 117); a finger phalanx from Denisova Cave, Russia (80, 97); and three nondiagnostic bone fragments (10). These remains range in age from approximately 72 to 200 ka (10, 32, 55, 94), suggesting a long, continuous occupation of the cave or multiple periods of occupation (106). Additionally, a mandible from Baishiya Karst Cave, Xiahe, Gansu, China, dated to 160 ka, has been attributed to this lineage by proteomic analysis (16). Finally, two crania and three additional pieces from Lingjing, Xuchang, China, are dated between 105 and 125 ka and exhibit a mosaic of ancestral and derived morphological features that encompass the variation of early and modern humans and Neanderthals (67) and have been proposed to be Denisovan (40). Like Neanderthals, Denisovans interbred with modern human populations, and the genomic evidence of Denisovan ancestry in modern human populations points to a wider geographic and temporal range than can be inferred from fossils alone. Currently, admixture analyses of modern humans point to at least four Denisovan lineages that interbred with East Asians, Papuans (twice), and Philippine Negritos (11, 20, 54, 64, 80, 97, 105, 126). The most recent of these introgression events may have occurred as recently as approximately 21 ka (20, 54), indicating that Denisovans may have persisted longer than Neanderthals. Collectively, the fossil and genomic data point to a large Denisovan geographic range, spanning southern Siberia to Papua New Guinea, as well as a long temporal duration.

The occurrence of multiple, deeply divergent Denisovan and Neanderthal lineages across many habitats suggests that these lineages likely exhibited and maintained phenotypic variation, potentially due to local adaptation. Therefore, the available genomic data are best viewed within their local geographic and temporal contexts. However, even the multiple high-coverage Neanderthal genomes now available are not sufficient to fully assess polymorphism levels and population-specific genotypes and phenotypes.

## FOUR HIGH-COVERAGE ARCHAIC HOMININ GENOMES ARE AVAILABLE

Most autosomal genetic analyses of archaic hominins focus on one high-coverage Denisovan genome (80) and up to three high-coverage Neanderthal genomes (70, 94, 95). Both the Denisovan genome, Denisova 3, and one of the Neanderthal genomes, Denisova 5, were sequenced from bones recovered in Denisova Cave in the Altai Mountains in southern Siberia that were dated to 72 and 122 ka, respectively (80, 94, 95). A second Neanderthal genome, Chagyrskaya 8, is from remains discovered in Chagyrskaya Cave, also in the Altai Mountains, that were estimated to date between 60 and 80 ka (70). Hereafter, we refer to the Denisova 5 individual as the Altai Neanderthal. Finally, a third Neanderthal genome, Vindija 33.19, was sequenced from remains found in Vindija Cave, Croatia, dated to 52 ka (94).

In addition to these four high-coverage genomes, there are a larger number of low-coverage sequences. Sequences from 14 Neanderthals span both the space (Gibraltar to Siberia) and time (39 to 120 ka) of Neanderthal European habitation (6, 44, 47, 88). All four high-coverage and several of the low-coverage genomes are of female individuals; however, at least two Y chromosome sequences are available for each lineage (86). Mitochondrial DNA sequences are also available for at least 18 Neanderthals (70, 91) and 7 Denisovans (10, 117). Most recently, nuclear DNA has been enriched from cave sediments and used to expand our understanding of Neanderthal population radiations and replacement (127).

These genomic sequences have enabled the characterization of individuals who could not be assigned to a lineage or population based on morphology alone. Denisovans were only identified as a separate lineage from Neanderthals based on sequence analysis (97). Furthermore, a first-generation hybrid, Denisova 11, who had a Denisovan father and a Neanderthal mother, was identified from bone found in Denisova Cave (116). Finally, analyses of both nuclear and mitochondrial DNA have shed light on archaic hominins from Sima de los Huesos, Spain. These individuals are Neanderthal-like in morphology, and their nuclear DNA is more similar to Neanderthals’ than to Denisovans’ (78), yet their mitochondrial DNA is closer to Denisovans’ than to Neanderthals’ (79). Sequencing and comparison of mitochondrial and nuclear DNA from additional early Neanderthals revealed evidence of deeply divergent mitochondrial DNA, suggesting complex population histories with substantial admixture between ancestors of Neanderthals, Denisovans, and modern humans (88, 91).

These genomic sequences allow for the identification of genetic differences between archaic hominins and present-day humans. In the following sections, we describe a range of approaches for translating individual (or, in some cases, many) genetic differences into phenotypic insights (Figure 1).

## PREDICTING EFFECTS OF GENETIC DIFFERENCES BETWEEN ARCHAIC HOMININS AND MODERN HUMANS

### Phenotypic Effects of Protein-Coding Differences

Protein-coding sequences are often under strong purifying selection and conserved among closely related species, including between archaics and humans. Furthermore, our understanding of how changes at the nucleotide level influence protein sequence, structure, and function is more mature than it is for other parts of the genome. Therefore, because of their functional importance and relative ease of interpretation, protein-coding variants have seen considerable attention in the inference of potential phenotypic effects of genetic differences between modern and ancient individuals.

The sequencing of four archaic genomes and many more exomes has enabled initial estimation of the number of fixed protein-coding differences between modern humans and archaics. These differences can range from large-scale copy number variants and segmental duplications to shorter insertions and deletions (indels) and single-nucleotide variants (SNVs). SNVs are the simplest form of protein-coding difference and the most straightforward to identify; 802 such differences (missense, nonsense, and synonymous) are fixed in genes among modern humans and two archaic genomes (Denisovan and the Altai Neanderthal) (95). The most disruptive fixed derived SNVs in both the Denisovan and the Altai Neanderthal are stop gains in *LASP1*, *OR5AC2*, and *RPL28* (95). An even smaller number of differences in fixed indels (<12 base pairs) occur within genes (*N* = 14), although there are many more in the noncoding portion of the genome (*N* = 37,179) (95). Finally, no segmental duplications are shared between Denisovans and Neanderthals relative to modern humans, but each archaic lineage exhibits specific segmental duplications: 8 and 6, respectively (95).

Despite the growing ability to infer the effects of a genetic variant on protein function itself, most previous work on protein-coding variants has not considered a variant’s effect on protein function beyond scoring its deleteriousness or describing its impact on splicing. Preliminary attempts to link protein-coding variants to phenotypic differences focused on the annotated functions of individual genes with protein-coding differences (Figure 1). This approach assumes that if genes involved in a biological function have genetic differences between archaic hominins and humans, then phenotypes related to the function are likely to have been different. It is often expanded to multiple genes with differences by testing for enrichment of specific functional annotations from organized ontologies, such as the Gene Ontology or Human Phenotype Ontology. However, the relatively small number of protein-coding differences between humans and archaic hominins limits the power of this approach. For example, Castellano et al. (14) analyzed three Neanderthal exomes and assessed enrichment in amino acid changes for each phenotype category from the Human Phenotype Ontology while correcting for gene size and base composition. They reported an overrepresentation of changes in genes related to metabolism, the cardiovascular system, hair distribution, and skeletal morphology compared with the ancestors of Denisovans and Neanderthals (14). Along the Neanderthal lineage, the only overrepresented human phenotype was hyperlordosis, which is consistent with morphological evidence that Neanderthals exhibited reduced lumbar lordosis compared with other Pleistocene hominins and modern humans (42).

In cases where the archaic allele is segregating in modern human populations—often due to introgression—it is common to leverage trait associations, such as those identified by genome-wide association studies (GWASs) or phenome-wide association studies (PheWASs), in human populations to infer archaic phenotype. For example, SNVs in pigmentation-associated genes suggest that Denisovans exhibited dark skin pigmentation, brown eye pigmentation, and dark hair color (15, 80). As another example, Neanderthals and Denisovans lack a stop codon in *CASP12*; this allele has been associated with increased risk of sepsis in humans (95).

As we describe in subsequent sections, the GWAS/PheWAS approach is commonly used to interpret other types of genetic variation (e.g., non-protein-coding) between archaic hominins and modern humans. However, this approach does not account for the genetic or environmental context of the archaic allele, limiting confidence about inferences of archaic phenotypes. Furthermore, this approach is limited to variants that are polymorphic in humans; such approaches cannot be used for alleles that do not occur in modern humans.

**Figure 2:**
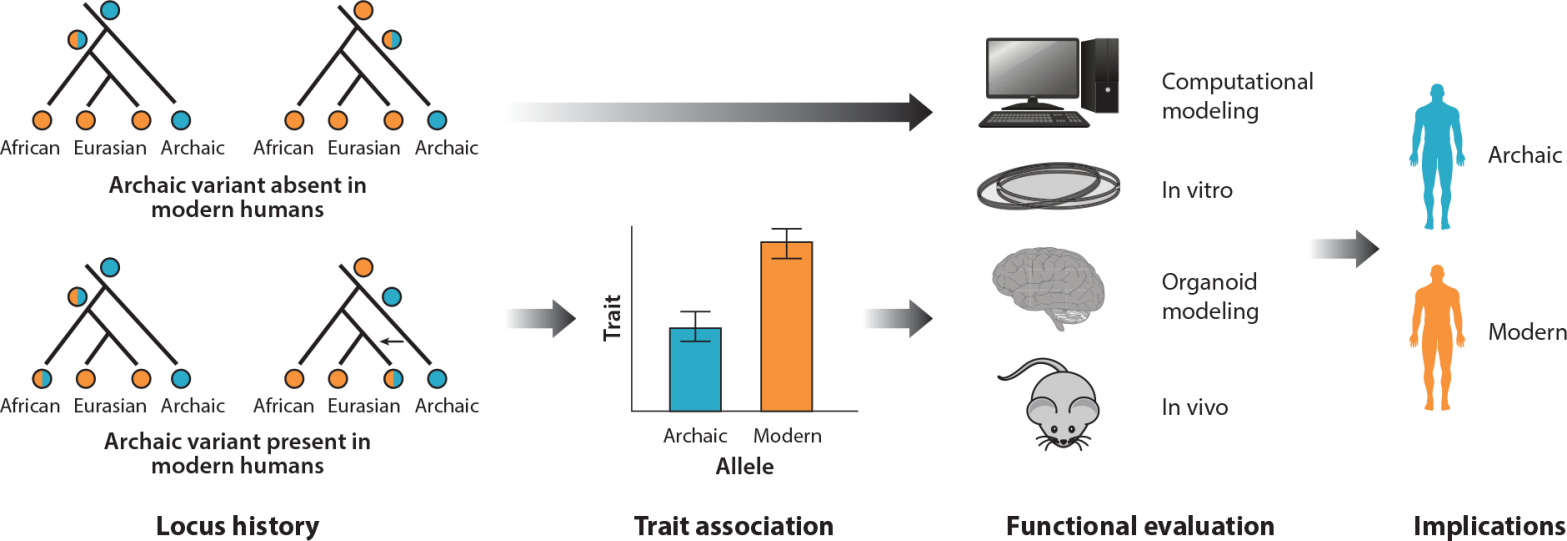
Steps in assessing and validating phenotypic effects of specific archaic alleles (*blue*). Based on the distribution of alleles across populations (i.e., the locus history), different approaches are possible. For archaic alleles that are segregating in modern humans (example histories at *bottom left*), potential archaic phenotypes can be inferred from traits that associate with the variant in modern humans. These associations can be validated using methods that speak to similarities and differences between archaic and modern human phenotypes. Archaic alleles that do not occur in modern humans (example histories at *top left*) cannot be associated with a trait and must be functionally evaluated in model systems. Blue circles represent alleles present in archaic individuals, and orange circles represent modern alleles.

Once a phenotype has been proposed for a protein-coding variant, several approaches can be used to functionally evaluate the effects (Figure 2). For example, Trujillo et al. (122) recently studied a protein-coding variant in *NOVA1* that is fixed in modern humans and emerged after the divergence from archaics. *NOVA1* regulates alternative splicing in nervous system development. The authors used CRISPR-Cas9 to introduce the archaic variant into human induced pluripotent stem cells and derived cortical organoids. This variant resulted in differences in gene expression, organoid morphology, cell proliferation, and synaptic protein interactions. Maricic et al. (72) suggested, however, that these differences may be driven not by the archaic allele itself but rather by deletions unintentionally introduced by the use of the CRISPR-Cas9 method. While the ultimate cause of the observed phenotypes and gene expression differences remains unclear, approaches that directly model an archaic allele’s effects in tractable cellular and organoid models show promise.

### Phenotypic Effects of Structural Variants

Identifying and assessing the phenotypic consequences of structural variants is difficult. This is especially true for deletions, which can lead to gene loss, fusion transcripts, or loss-of-function alleles if they occur in protein-coding regions (68). Thus, purifying selection is more likely to remove such deletions from a population, which limits our ability to study them. In addition, due to their size, structural variants can influence multiple genes and have a large impact on phenotypes (34). Efforts thus far have focused on interpreting the functions of genes included in the structural variants. Meyer et al. (80) reported two Denisovan-specific duplications: one on chromosome 3 that was 31 kb in length and did not span any known genes, and another on chromosome 4 that was 43 kb in length and overlapped *UBA6* and *LOC550112*.

Prüfer et al. (95) identified segmental duplications in the Denisovan and Altai Neanderthal. Surprisingly, they found no duplications that were shared across the sequenced archaic hominins. Denisovan duplications overlapped the genes noted above as well as *KIR2DL4*, *KIR3DP1*, *LOC100131199*, *LOC550112*, *MPZ*, and *SPATS2*, whereas the Neanderthal duplications overlapped *MMP17*, *ORA11*, *RAF1*, and *ULK1*. Inferring the phenotypic consequences of these duplications is difficult because such events are typically thought to result in changes of function (often loss of function) in at least one of the copies (22). Additionally, the limited number of genes in Denisovan or Neanderthal segmental duplications limits the utility of existing functional annotations.

Indels that modify smaller genomic regions (typically 1–100 base pairs) are another important class of structural variation that can impact portions of genes, entire genes, or regulatory elements. Work to date has focused almost entirely on deletions. Prüfer et al. (95) identified 212 regions totaling 1.5 Mb of deleted sequences in archaics. Only a small fraction of these losses were shared across both lineages: 35 regions spanning 141,723 base pairs. These regions overlapped four loci: *C11orf36*, *GSTT1*, *LOC391322*, and *MRGPRG*. Denisovans also completely lost *LCE3C*/*LCEB*, whereas Neanderthals completely lost *TFAMP1*.

The effects of archaic indels that are polymorphic in modern humans can be explored by associating the locus with phenotypes in extant human populations (Figure 2). However, this approach can only be used for the handful of archaic indels observed in humans. Chintalapati et al. (19) studied the impact of introgressed coding and noncoding indels on human phenotypes and found nine such loci that were associated with multiple phenotypes: age-related macular degeneration, cleft lip, menarche, type 2 diabetes, suicide attempts in bipolar disorder, preeclampsia, and insulin-related traits. Carriers of the introgressed indel associated with menarche exhibit earlier menarche compared with noncarriers. Lin et al. (68) examined 17 deletions shared between either or both archaics and humans, including four that result in a loss of function. Among these four is a deletion in an aforementioned gene: *LCE3C*. The *LCE3C* deletion is shared between modern humans and Denisovans, has been associated with increased risk of psoriasis, and occurs at high frequencies in Eurasians. A deletion encompassing *UGT2B28*, involved in steroid metabolism, is shared with Neanderthals and occurs at moderate frequencies in Africans. Another shared Neanderthal deletion occurs in *ACOT1*. This gene may be involved in the regulation of milk fat synthesis, and copy number variants near this locus were recently proposed to be adaptively introgressed among Melanesians (51). Only one loss-of-function deletion, in the spermatogenesis-associated gene *SPATA45*, was determined to be introgressed from Neanderthals.

### Phenotypic Effects of Differences in DNA Methylation

Despite the previous focus on protein-coding changes, the vast majority of genetic differences between modern human and archaic hominins are in noncoding regions of the genome (62, 80, 95). While it is relatively straightforward to identify which gene a protein-coding nucleotide change affects and thereby draw some conclusions about its influence on protein sequence and structure, linking noncoding changes to function is more challenging. Many functional noncoding genetic variants influence gene regulation, and these changes are thought to have a major influence on complex trait variation and evolutionary divergence between closely related species (61). However, gene regulation is a complex and dynamic process that involves the accessibility of DNA, the binding of transcription factors to specific DNA sequences, interactions of accessory proteins that recruit the transcriptional machinery, a wide range of chemical modifications to DNA and histone proteins, and the three-dimensional structure of the genome itself. Genetic changes to regulatory regions, such as promoters, enhancers, and insulators, can influence the expression patterns of genes megabases away, and identifying where these regulatory regions are in the genome and the genes they influence is an ongoing focus of genomics research (82).

DNA methylation at CpG dinucleotides is associated with gene expression and is dynamic across cellular states. As a result, many methods have been developed to identify methylation at CpGs genome-wide. Methylation at a locus is associated with an inactive regulatory state, and the absence of methylation is often indicative of regulatory activity. Variation in methylation between individuals and in different cellular states can thus indicate differences in gene expression. Accordingly, epigenome-wide association studies have revealed that differentially methylated regions (DMRs) play a role in development (118) and have been associated with multiple phenotypes, including some diseases (36). Unlike other epigenetic marks, DNA methylation can be measured directly from DNA (rather than from the histone proteins it is wrapped around) and is therefore potentially measurable in studies of ancient DNA (75). Once DNA methylation has been measured, regions of the genome that are likely to be regulatory—both genic promoters and more distal elements—can be identified and used to compare between modern humans and archaic hominins. If these differences can then be attached to specific genes, many of the same annotation-based analysis strategies described for protein-coding changes can be applied.

DNA methylation can be measured from ancient DNA both directly and indirectly (75). The direct approach employs bisulfite sequencing, which capitalizes on the different reactions of methylated and unmethylated cytosines to sodium bisulfite and can capture methylation at the level of an individual base pair. While this method works well for present-day samples, ancient samples are often fragmented and may be considerably deaminated, limiting the application of bisulfite sequencing only to well-preserved ancient samples (108).

**Figure 3:**
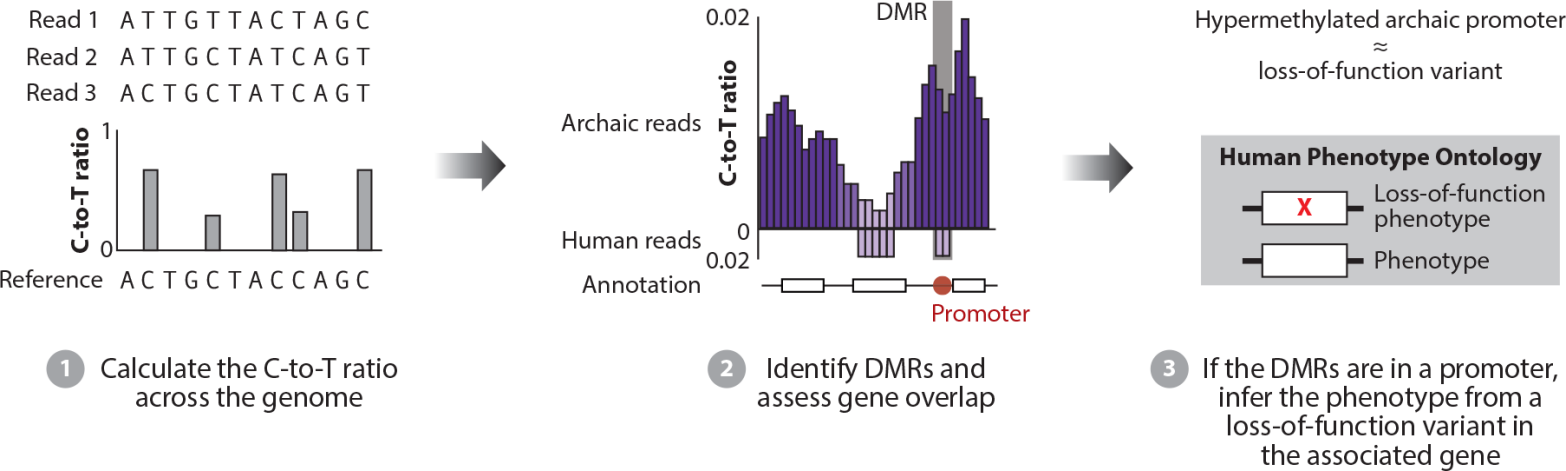
Estimating phenotypes from DMRs. The ratio of cytosines to thymines is calculated per base pair using a reference genome. DMRs between a target population (e.g., archaics) and a reference (e.g., humans) can be identified by divergent patterns at a locus. For genes with differences in promoter methylation, phenotypic effects can be estimated based on gene annotations, e.g., by comparing with loss-of-function variants in the gene or associated phenotype from an ontology (e.g., the Human Phenotype Ontology). Abbreviation: DMR, differentially methylated region.

The indirect approach to assessing DNA methylation in ancient samples is based on the observation that over time, methylated cytosines decay into thymines, whereas unmethylated cytosines decay into uracils (75). As ancient DNA sequencing protocols remove uracils, methylated regions should have a higher proportion of thymines compared with unmethylated regions (Figure 3). Gokhman et al. (39) used this approach to generate regional methylation maps for the Denisovan and Altai Neanderthal genome. Given that methylation is dynamic across cell types, these maps reflect the state in the source bone tissue. It remains unclear to what extent these data are relevant for phenotypes involving other tissues. Comparing these maps with those from modern human osteoblasts identified approximately 1,100 DMRs, of which 295 were Denisovan specific and 307 were Neanderthal specific. The *HOXD9* promoter and *HOXD10* gene body were hypermethylated in both the Denisovan and the Neanderthal genomes, and the *HOXD9* gene body was hypermethylated in the Denisovan genome (39). This study also found that changing an individual transcription factor could potentially impact multiple DMRs and that genes with DMRs were twice as likely to be related to disease (39).

Given the near absence of data on Denisovan skeletal anatomy, Gokhman et al. (40) used archaic methylation maps to predict anatomical phenotypes from DMRs. The authors considered only DMRs within 1–5 kb of a transcription start site, genes whose promoter had a DMR, and genes that affect bones or teeth, given that the archaic DMR data come from genomes generated from bone samples (Figure 3). To validate their approach, they predicted anatomical traits using the same methylation-based approach for Neanderthals and chimpanzees, two species with at least some phenotypic data. Some anatomical traits could be predicted from DMRs with high precision and sensitivity in these taxa. Applying this method to Denisovans revealed anatomical phenotypes shared with Neanderthals, including a robust jaw, a low cranium, increased cranial base growth, a low forehead, thick enamel, a wide pelvis, large femoral articulations, wide fingertips, and a large ribcage. There were three unique Denisovan traits that were not shared with Neanderthals and modern humans: an elongated dental arch, an enlarged mandibular condyle, and biparietal expansion.

Studies of phenotypic changes associated with differential methylation in modern humans are important to evaluate the efficacy of such approaches and their application to archaics. Archaic methylation maps are also useful for studying human-derived traits that emerged after our divergence from the Neandersovans. For example, differentially methylated genes between humans and archaics are enriched in GWAS SNVs for schizophrenia and potentially height (3). Human DMRs are also enriched for genes that affect facial and vocal anatomy, hair, and the spinal column (4, 41).

### Phenotypic Effects of Differences in Gene Expression

Analysis of differences in transcription levels as measured by RNA sequencing provides a powerful way to understand phenotypic differences in modern populations (45). Large-scale functional genomics projects like the Encyclopedia of DNA Elements (ENCODE) and the Roadmap Epigenomics Project have created catalogs of regions of the genome with potential regulatory functions (33, 63).

It would be ideal to study gene expression differences between humans and archaic hominins directly, as has been done for larger evolutionary distances between extant species (9, 13, 17, 59). However, in the case of ancient individuals, measuring RNA remains an impossibility. Thus, we must infer effects on regulation from sequence differences.

Several approaches have focused on intersecting individual genetic differences with gene regulatory elements identified in humans and testing for enrichment or depletion. For example, Telis et al. (120) studied the regulatory effects of introgressed archaic variants by intersecting them with enhancers from the Roadmap Epigenomics Project. They showed that enhancers, particularly in the brain, were depleted of Neanderthal and Denisovan alleles, suggesting that archaic alleles in those contexts likely produced different phenotypes in humans that drove purifying selection. Similarly, Silvert et al. (109) also looked at introgressed variants in annotated enhancers, as well as promoters and microRNAs. They also observed a depletion of archaic alleles in regulatory regions; moreover, they found an enrichment of archaic alleles at a very high frequency (>95% minor allele frequency) in enhancers, suggesting that in some cases regulatory effects could have contributed to adaptation.

While studying variant overlap with regulatory regions can help identify variants likely to influence phenotypes, the difficulty in linking affected regions to the expression of specific genes remains a significant impediment to identifying the affected phenotypes (37). Paired RNA-sequencing and genotype data collected by the Genotype-Tissue Expression (GTEx) Consortium (45) have become a valuable resource for identifying both regulatory variants and the genes they influence. With sample counts in the hundreds for nearly 50 different tissues, this data set is by far the most comprehensive for studying regulatory variants and their effects on specific genes. Most notably, the GTEx Consortium identified millions of variants that are correlated with the expression levels of specific genes [expression quantitative trait loci (eQTLs)], allowing observed genetic variation to be connected to effects on specific genes and thereby to downstream phenotypes.

By intersecting Neanderthal introgressed alleles with GTEx eQTLs, Dannemann et al. (28) identified a set of archaic alleles that influence gene expression in Europeans. They found that Neanderthal alleles are more likely than non-Neanderthal loci to be associated with differential gene expression. The loci also overlap with GWAS hits for metabolic pathways, immunity, and neurological phenotypes, indicating that these Neanderthal regulatory variants may also influence organism-level phenotypes. For example, the authors identified a Neanderthal allele associated with decreased expression of IL18 that is also associated with altered inflammatory response in several GWASs. McCoy et al. (77) went a step further by identifying cases of Neanderthal allele-specific expression (i.e., different allelic expression levels for transcripts with and without Neanderthal alleles in heterozygous individuals). Genes exhibiting allele-specific expression included *TLR1*, *SLC15A4*, and *ADAMTSL3*, and Neanderthal alleles were significantly downregulated in testes and brain tissues. Many of the highlighted variants were associated with diseases, indicating that these expression changes likely affect phenotypes. However, Rinker et al. (98) identified that many introgressed haplotypes carry functional ancestral alleles that were lost in some non-African populations. Many of these alleles influence gene expression in modern humans, and introgression reintroduced thousands of such alleles (98); thus, some of the effects of introgressed haplotypes on expression may not be due to Neanderthal-derived alleles. For this reason, and those previously raised, resolving the specific causal variants underlying the regulatory effects of introgressed haplotypes and the implications for phenotypes of archaic hominins will require further integration of sequence analysis and experimental validation.

**Figure 4:**
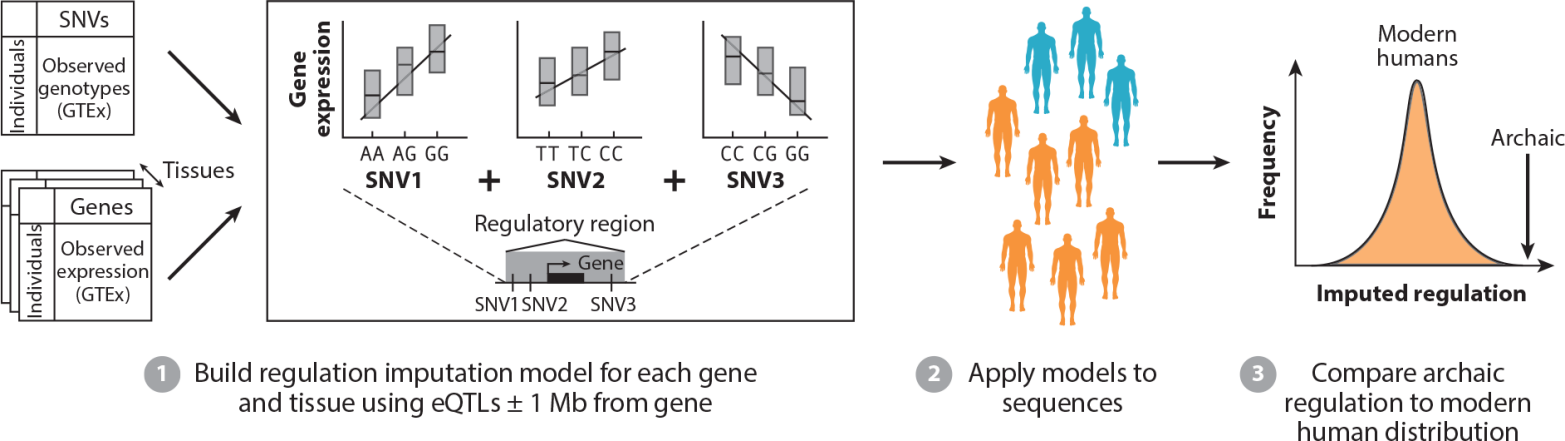
Estimating divergent gene regulation in archaics. Models for imputing the genetic component of gene expression are constructed for each gene using common SNVs located within 1 Mb up- or downstream and expression data from diverse tissues from GTEx. These models can then be applied to sequences from modern humans and archaics. Divergent regulation is suggested when an archaic individual falls outside the range of the modern humans. Abbreviations: eQTL, expression quantitative trait locus; GTEx, Genotype-Tissue Expression; SNV, single-nucleotide variant.

While we cannot directly observe archaic hominin gene expression, recent machine learning methods show promise for predicting the effects of variants on gene expression. Colbran et al. (21) applied one such method, PrediXcan, to predict regulatory differences between archaic hominins and modern humans genome-wide, including regions that do not retain Neanderthal ancestry. PrediXcan is trained on GTEx data to build regression models for predicting the genetic component of the regulation of each gene (analogous to eQTL studies but modeling multiple variants at once) (Figure 4). We identified 2,290 genes whose imputed regulation for the Denisovan and the Altai and Vindija Neanderthals fell outside the variation observed in modern humans, 766 of which were in regions of the genome that do not retain Neanderthal ancestry in modern populations. These genes are involved in diverse disease traits, such as spontaneous abortion, polycystic ovary syndrome, myocardial infarction, and melanoma. Linking the genes divergently regulated between modern humans and archaic hominins to phenotypes from the Human Phenotype Ontology revealed many genes involved in skeletal and dental morphology and skin pigmentation (21).

These methods of studying regulatory differences between humans and archaics have enabled great strides in identifying genes and phenotypes that likely differ between groups. However, there are still areas for future work. For example, all of the current eQTL-based studies are based on GTEx data, which consist primarily of common variants identified from individuals of European descent. This means we lack direct evidence of regulatory activity, not only for non-introgressed variants, but also for variants that are rare or specific to other populations with archaic ancestry. This is most notable for Denisovan-specific variants. Similarly, it is unclear to what extent the overrepresentation of European ancestry in annotation projects such as the Roadmap Epigenomics Project affects the identification of regulatory regions. In addition, while these methods can analyze multiple archaic variants together, they do not model the full archaic genomic background. Thus, as mentioned previously, an effect seen for archaic variants in a human context would not necessarily be the same if the variant were embedded in a full Neanderthal genome.

### Phenotypic Effects Based on Statistical Associations with Genotypes

Rapid increases in the amount of available genomic and phenotypic data from thousands of individuals have enabled analyses that aim to statistically link single alleles or combinations of alleles to phenotypes without considering the molecular mechanisms by which the variants function. These approaches are agnostic to whether variants occur in coding or noncoding regions of the genome. Such studies are important for understanding the impact of introgression on human health and other traits, but also provide insight into archaic phenotypes themselves.

Sankararaman et al. (104) described associations between Neanderthal alleles and several human phenotypes based on previous GWASs: autoimmune disease (Crohn’s and lupus), biliary cirrhosis, IL18 levels, optic-disk size, smoking behavior, and type 2 diabetes. Simonti et al. (110) integrated electronic health records with genome-wide genotypes and Neanderthal haplotypes inferred by Vernot & Akey (124). Based on genome-wide complex trait analysis, Neanderthal alleles collectively explained significant but small levels of risk for eight phenotypes in two cohorts from the Electronic Medical Records and Genomics (eMERGE) Network: actinic keratosis, acute upper respiratory infections, coronary atherosclerosis, depression, obesity, being overweight, mood disorders, and seborrheic keratosis. Additionally, a PheWAS identified four SNVs directly associated with specific phenotypes: hypercoagulable state, protein-calorie malnutrition, urinary system symptoms, and tobacco use. Dannemann & Kelso (27) conducted a similar analysis using data from the UK Biobank, which included phenotype data on both disease- and non-disease-related traits. They used Neanderthal alleles determined by Sankararaman et al. (104) and found associations with multiple phenotypes: chronotype, comparative height at 10 years of age, ease of skin tanning, hair color, impedance of right and left legs, incidence of childhood sunburn, narcolepsy, pulse rate, sitting height, and skin color (27).

Most recently, McArthur et al. (76) leveraged linkage disequilibrium score regression methods (35) to estimate the contribution of alleles introgressed from Neanderthals to the heritability of more than 400 human traits. As expected, most traits were significantly depleted for contributions from introgressed alleles, but eight traits exhibited significant heritability enrichment in Altai-matching introgressed variants: autoimmune disease, forced vital capacity, heel T score, menopause age, being a morning person, vulnerability to sunburn, type 1 balding, and white blood cell count (76).

Multiple studies have focused on specific categories of phenotypes or specific phenotypes themselves. Neanderthal alleles have been associated with reduced endocranial globularity (46), pain sensitivity (137), reproduction (138), oxidative stress (23), innate immune response (24, 30, 102), coronavirus disease 2019 (COVID-19) risk (139), and COVID-19 protection (140). Health-related phenotypes that can be derived from genomic data are of immediate interest because they can be used to estimate when these phenotypes emerged in humans and can speak to the evolutionary history of those traits. Estimating these traits in the Altai Neanderthal is of particular interest because her genome shows evidence of inbreeding (95), which could have deleterious health consequences.

Polygenic risk scores (PRSs), which statistically integrate variant effects estimated from GWASs to predict phenotypes based on genotypes, are becoming common in human genetics (121). One recent study predicted disease burden and calculated PRSs for nine disease categories in ancient humans and archaics (5). The Altai Neanderthal was predicted to be in poor genomic health and have high risk for cancer, gastrointestinal and liver diseases, immune system–related diseases, metabolism-related disorders, neurological disorders, and morphological and muscular diseases. By contrast, this individual had low risk for cardiovascular disease and average risk for periodontal and miscellaneous disease. The Denisovan individual was also predicted to have high risk for immune system–related diseases. However, given the challenges of building PRSs that generalize across modern human populations (73, 83), it is not clear how accurate the models’ predictions are for the archaic individuals.

Direct association of genotypes and phenotypes is a powerful approach for generating candidate phenotypic differences and causal loci. However, the associative nature of these studies requires further evidence for causality from functional studies (Figure 2), especially given that many loci may be linked to nearby nonarchaic variants (98, 114) or are pleiotropic (112, 119). Study designs for GWASs primarily prioritize limiting false positives, with strict corrections for multiple hypothesis testing (58), which suggests that some phenotypic associations for archaic variants may be missed given available sample sizes and allele frequencies. However, even when it is difficult to pin down the precise biological mechanisms driving an association, these methods remain powerful for identifying traits and organ systems that are likely to have differed in archaic hominins.

## WHAT DO WE KNOW ABOUT ARCHAIC HOMININ PHENOTYPES FROM GENOMIC DATA?

**Figure 5:**
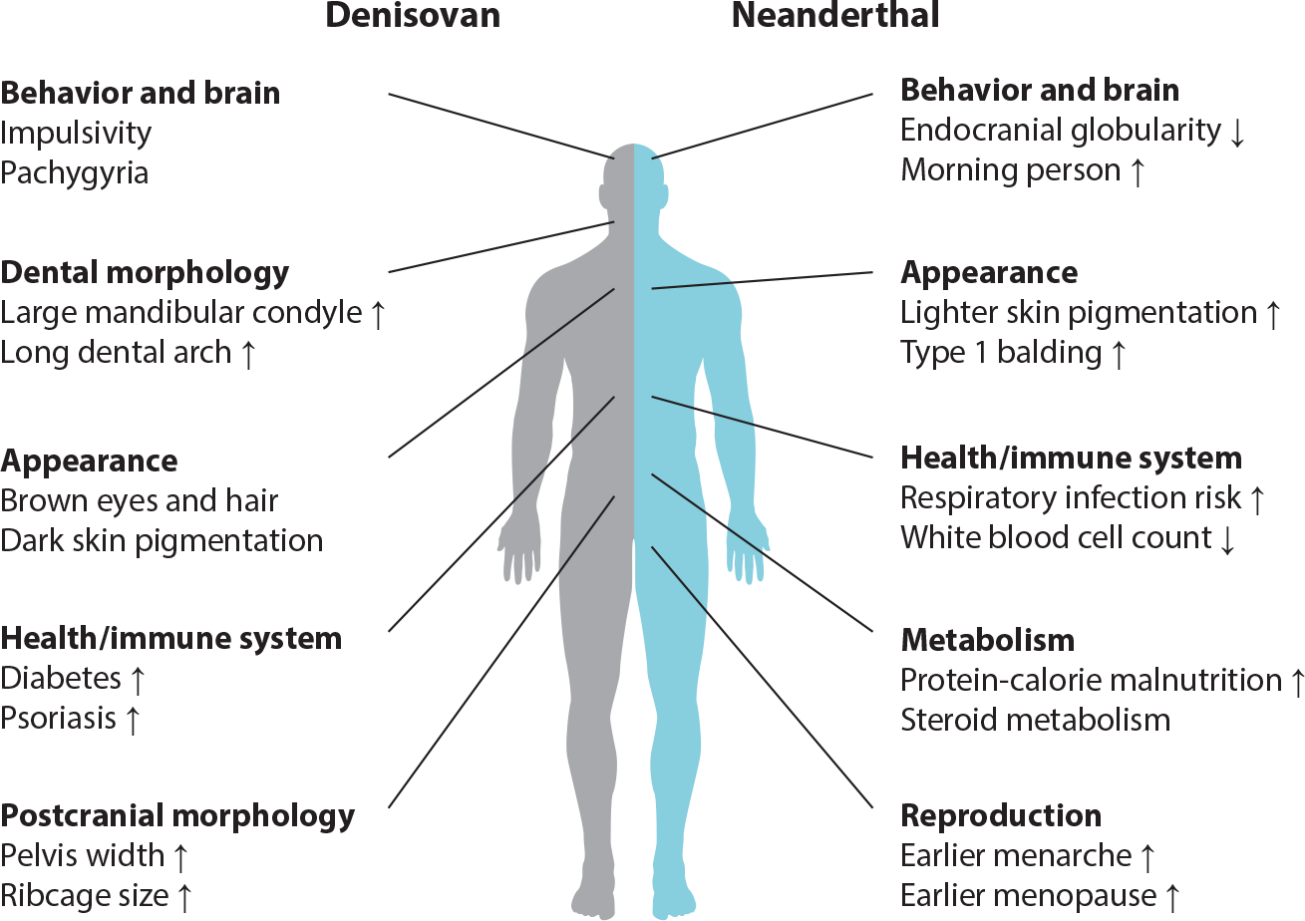
Modern human phenotype categories and example phenotypes that have been proposed to be associated with Denisovan and Neanderthal genetic variation. Arrows indicate the directionality of the trait difference when known.

In this section, we briefly summarize what analyses of genomic data have revealed about phenotypes that have been influenced by genetic differences between archaics and modern humans. Figure 5 highlights broad themes that have emerged from these studies and a few example traits. We also refer readers to Supplemental Tables 1 and 2 for a more complete list of these phenotypes.

Studies of variants in protein-coding regions have largely bypassed quantifying differences on protein function and relied on associations between the variants and phenotypes in modern humans to infer Neanderthal phenotypes. Many of these associations are related to morphology (e.g., hypolordosis) (14). Other protein-coding differences point to potential differences in physiology (68) and possibly the brain (122). Neanderthal DMRs are near genes related to the immune system and skeletal morphology (39). Furthermore, phenotypic inference from these DMRs confirmed or extended much of what is known about Neanderthal skeletal morphology from the fossil record (40). Inferences of Neanderthal gene expression point to divergent phenotypes from modern humans in many categories, including the brain, the immune system, morphology, physiology, and reproduction (21, 77). Finally, most of our understanding of Neanderthal phenotypes comes from studies that use association analyses to directly link variants to phenotypes. Collectively, this research points to differences in appearance, behavior, the brain, immune function, morphology, physiology, and reproduction compared with modern humans (27, 46, 76, 104, 110, 114, 137–140).

Variants in Denisovan genes have emphasized phenotypes related to appearance, behavior, the brain, and physiology (15, 68, 80, 114), while DMRs may provide insight into immune function and skeletal morphology (39, 40). Potential differences between Denisovans and modern humans in these categories are further supported by evidence of divergent regulation in the genes underlying these phenotypes (21).

## MOVING FORWARD

Equipped with millions of modern human genomes, genotypes for thousands of ancient humans, and several archaic hominin genomes, what impedes us from inferring more about archaic hominin phenotypes? In our view, there are two fundamental challenges: (*a*) the limited availability of genomic data from diverse modern and archaic individuals and (*b*) the difficulty of accurately predicting phenotype from genotype.

### Challenge 1: Limited Availability of Diverse, High-Quality Archaic and Modern Genomes

Additional archaic hominin genomes, particularly from currently unsampled regions in their expansive geographic ranges, would be tremendously useful in understanding their phenotypic variation and history. However, in addition to the difficulty of finding potential samples, it is challenging to recover DNA from specimens from many relevant locations, such as sub-Saharan Africa and tropical Southeast Asia, due to environmental conditions that do not preserve DNA well. Nonetheless, the sequencing of additional genomes, especially from Denisovans, will be essential to assessing archaic hominin genetic and phenotypic variation.

There is also a need for more and higher-quality genomic data from humans. Even though genome sequencing is now common, available modern human sequences are substantially biased toward individuals of Eurasian ancestry (12, 89, 111). The human genetics community has acknowledged this issue, and several efforts are underway to increase diversity (81, 141). Ancient DNA from humans is beginning to enable researchers to trace the evolution of human-specific phenotypes and clarify relationships among genotype, phenotype, and the environment, but the available sequences are also substantially biased toward individuals from Europe and central Asia due to both technical and social factors (69, 71).

Another challenge is that the most widely used human reference genome was only 92% complete and was missing many regions, including complex rearrangements, the entire p arms (short arms) of the five acrocentric chromosomes, and large human satellite arrays (85). A nearly 100% complete reference genome was introduced in mid-2021, adding 182 Mb of novel sequence that covers 115 predicted protein-coding genes (85). While much of this sequence is highly repetitive and thus may be difficult to map in all but the highest-coverage ancient genomes, additional differences between modern humans and archaics that are relevant for various phenotypes may lie within these regions.

### Challenge 2: Difficulty of Accurately Mapping Genetic Variation to Phenotypic Variation

Our limited understanding of the complex mapping between genotype and phenotype is a substantial barrier to determining physical attributes of archaic groups. While some phenotypes, such as Mendelian diseases, have relatively simple genetic architectures, most traits of particular interest to evolution and anthropology do not. Human complex traits have (*a*) many associated or causal loci with weak or modest effect sizes and (*b*) causal loci that are spread across the genome. Furthermore, most variants likely to influence differences in complex traits between humans and archaic hominins occur in noncoding regions rather than protein-coding regions. Moreover, for many traits, rare genetic variants (frequency <1%) make substantial contributions to phenotypic variation (7, 49, 130). These observations, paired with the complex molecular networks linking genes’ functions, prompted the proposal of an omnigenic model for human phenotypes (8). This model posits that additive effects on a phenotype are partitioned into effects on core genes, which directly influence the phenotype, and effects on peripheral genes, which indirectly impact phenotypes through interactions with core and other peripheral genes (8). Mathieson (74) proposed that the omnigenic model explains why GWAS loci often replicate between populations but effect sizes do not. Therefore, even when accounting for differences in allele frequency and linkage disequilibrium, there is likely an upper limit to the transferability of genetics-based prediction strategies for complex traits, such as GWASs, PheWASs, and PRSs (74). Thus, the confident application of such methods in archaic hominins would require understanding when such methods fail to generalize, which requires increasing the diversity of genomic data sets of all kinds. Given the challenges of finding discovery data sets that match the ancestry and environment of archaic populations, we anticipate that the application of PRS-like approaches to archaic individuals may not be possible for many organism-level polygenic traits.

An additional complication when inferring phenotypes (especially in archaics) is accounting for the effects of the broader genomic and environmental context in which a variant of interest occurs. For example, many of the studies reviewed here have inferred archaic phenotypes from the phenotypic associations of introgressed variants in modern humans in modern environments. This approach assumes that other differences between human and archaic genomes do not substantially modulate the effect of the variant being studied (53). The differences in selection against introgressed variants in different gene regulatory elements (77, 120) suggest that the tolerance for archaic DNA in different functional contexts likely varied, with particularly strong intolerance of archaic variants in the brain. In addition, some traits in modern environments do not have clear analogs in archaic environments, e.g., addiction to specific substances or response to pathogens not present 40,000 years ago. These complications also apply to studies that introduce archaic variants into a human model system or cell line. The linking of genotypes to phenotypes is further complicated by the many nongenetic factors that can modulate the resulting phenotype. For example, deletion of the third exon in *GHR* is associated with malnutrition in humans and results in sexual monomorphism by weight and female-like liver gene expression under calorie restriction in mice (101). Such phenotypic variation from nongenetic factors is particularly evolutionarily relevant for understanding the phenotypes of archaics and modern humans because of the diverse and variable environments in which they lived.

These challenges underscore the need for studies that integrate computational and experimental approaches to simultaneously assess the phenotypic effects of multiple archaic DNA sequences in different contexts. While it is not feasible to consider an entire archaic genome at once, isogenic cell lines that have an archaic allele on both chromosomes for a given locus or at multiple loci in a functional pathway could help control for differences due to genetic background (26). Indeed, induced pluripotent stem cells show promise for understanding not only archaic phenotypes but also human evolution more broadly (25). Furthermore, the use of organoid models and CRISPR-Cas9 technology will be important for moving beyond trait associations to functionally validate archaic alleles (122).

### A Promising Approach: Inferring Molecular Phenotypes from Archaic Genomes

We argue that archaic hominin phenotypes can be much more confidently inferred when analyses are focused on lower biological levels, such as gene expression or protein structure. The mapping from genotype to these molecular phenotypes is less polygenic. In addition, while effect sizes and allele frequencies might vary between populations and environments, the underlying biology does not. A further advantage of this approach over methods that attempt to associate variants directly with organism-level phenotypes (GWASs, PheWASs, and PRSs) is the identification of testable hypotheses about the molecular mechanisms underlying functional differences, e.g., a change in protein stability or gene expression level in a specific tissue. Thus, we encourage future studies to focus on molecular traits and to use them as the basis for comparisons. It may then be possible to develop methods that combine information from these lower levels to make accurate predictions about organ- and organism-level differences.

Given the recent success of inferring archaic methylation and gene regulation, we believe that there are many promising avenues for future research on archaic molecular phenotypes. Powerful machine learning methods are being developed from massive human and model organism data sets to predict more and more cellular attributes relevant to function and disease from sequence information alone. These methods will enable studies based on protein structure and splicing, as well as larger-scale three-dimensional genome organization.

This review has focused on archaic hominins; however, the methods, limitations, and future directions covered here also apply to ancient DNA studies in other taxa. Indeed, such applications may provide important comparative information, particularly for other large-bodied mammals that inhabited similar environments as human ancestors. As ancient and modern genomic data sets increase in number and diversity, and as prediction algorithms based on those data improve, we will expand our understanding of the biological connection between genotypes and phenotypes.

## Supplementary Material

Supplementary Tables
